# Understanding Gender Wage Disparities in Dermatology

**DOI:** 10.2196/65003

**Published:** 2026-09-30

**Authors:** Danielle Duffle, Ramiro Rodriguez, Benjamin Brooks, Robert Dellavalle

**Affiliations:** 1 Department of Dermatology, OhioHealth Riverside Methodist Hospital, Columbus, OH, United States; 2 Department of Dermatology, Anschutz Medical Campus, University of Colorado School of Medicine, Aurora, CO, United States; 3 Department of Clinical Education, Rocky Vista University College of Osteopathic Medicine, Irvins, UT, United States; 4 Department of Dermatology, University of Minnesota School of Medicine, 1 Veterans Drive, Minneapolis, MN, 55417-2309, United States, 1 720-289-0247

**Keywords:** female physicians, women physicians, gender gap, dermatology, leadership in dermatology, pay disparities in medicine, nonlinear career trajectories, Claudia Goldin, Nobel Prize in Economic Sciences 2023, work-life balance in medicine, gender representation in dermatology, parental leave policies, women dermatologists, gender bias in medicine, career advancement for women physicians

## Abstract

Differences in compensation between men and women exist, despite having similar experiences. Here, we present data from the Association of American Medical Colleges (AAMC) from 2022 of self-reported compensation rates of academic dermatologists. Women dermatologists comprised 36.95% (75/203) of full professors, 61.54% (152/247) of associate professors, and 63.47% (311/490) of assistant professors; among these, 52.17% (12/23) were service chiefs and 35.48 % (22/62) were department chairs. The data shows that, except for department chairs, women are reporting being paid less relative to men at all academic ranks. Division chiefs had the most significant (45.52%) wage difference (45.52%) between men and women at US $281,674. This is a $281,674 difference between men and women chiefs per year. Women earned US $91,738.73 less on average per year when considering all academic positions. Different career trajectories and societal pressures to take time off for family-related absences are two of many factors contributing to the observed differences. We conclude that engagement at a national, institutional, and personal level is needed to better understand the observed differences, and future research should focus on how to improve it.

“Female physicians do the impossible” [1]. Claudia Goldin, awarded the 2023 Nobel Prize in Economic Sciences for her research on gender disparities in labor markets, provides an economic framework that helps explain gender-based differences observed within dermatology [1]. Goldin provides insights into women’s pay gap and leadership underrepresentation, which occurs despite more women entering dermatology (1013/1633, 62.03% in 2022‐2023) [2]. While female representation has improved, we hypothesized that disparities in pay within leadership positions persist despite increased female representation [3,4]. Using data from the Association of American Medical Colleges (AAMC), we illustrate leadership and wage differences between men and women in dermatology and analyze these trends within Goldin’s framework [3,5]. The purpose of this editorial is to help improve the understanding of the persistent differences between men and women in dermatology, despite similar academic rankings. Limitations of this analysis include its moment-in-time nature. Changes in salary trends could offer additional information on progress toward equity. Additionally, the AAMC did not include data on respondents’ age or years of experience within leadership roles.

Percentages of women in dermatology by academic rank, leadership roles, and salary were contrasted with those of men using data from the AAMC Faculty Salary Report 2022 [3]. *χ*^2^ analysis tested statistical significance (*P*<.05). Women comprise 36.95% (75/203) of full professors, 61.54% (152/247) of associate professors, 63.47% (311/490) of assistant professors, 52.17% (12/23) of chiefs, and 35.48 % (22/62) of department chairs (Figure 1). Division chiefs had a US $281,674.29 (45.52%) difference in mean income between female (–26.79%) and male dermatologists (+18.73%; Figure 2). Excluding Mohs training increased this gap to US $320,420.80.

**Figure 1.**
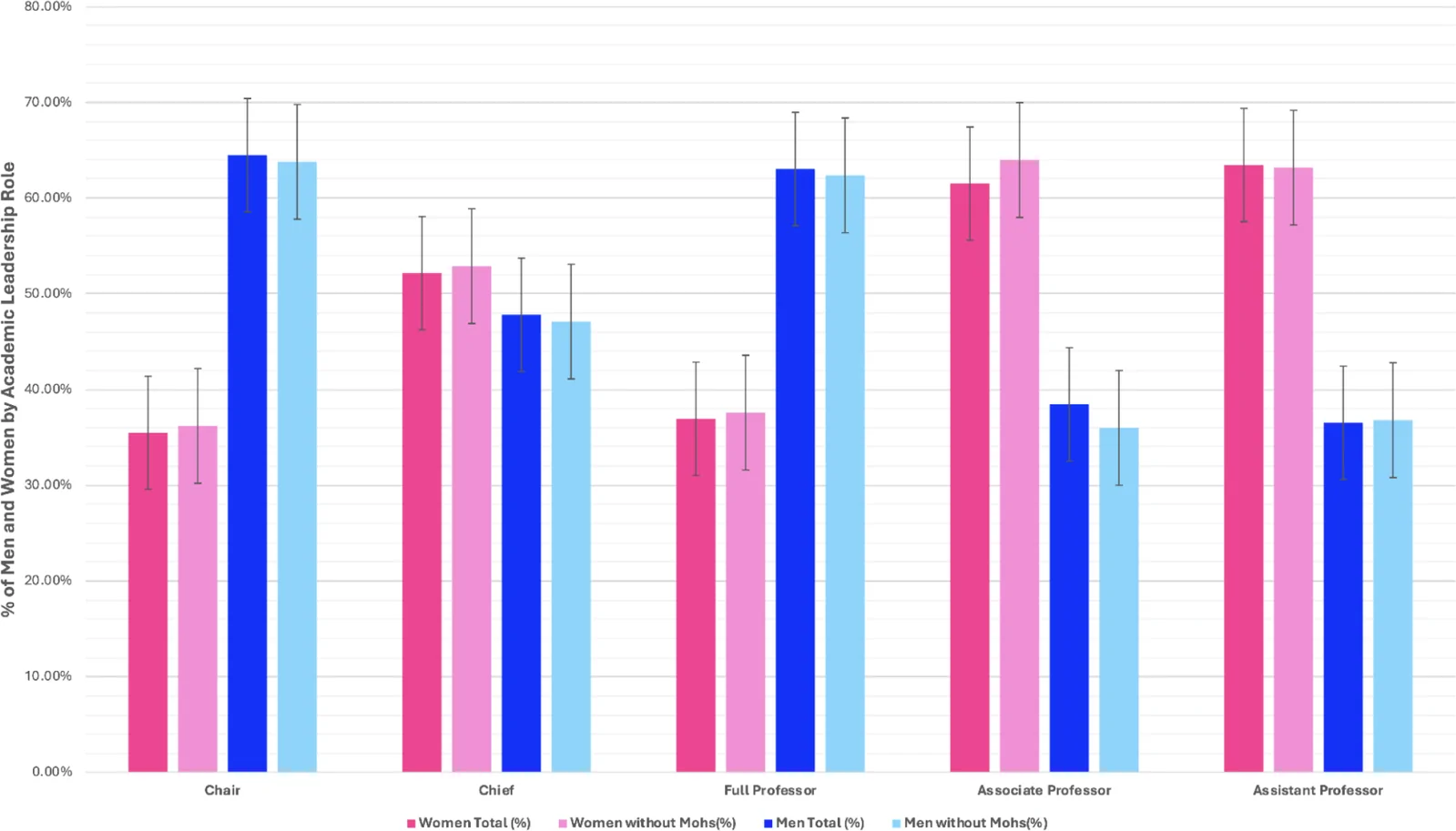
Percentage of women and men in dermatology academic leadership roles in the Association of American Medical Colleges (AAMC) Faculty Salary Report 2022 [3] (57.73%). Department chair salaries had a US $1505.33 difference (Figure 2). There were significantly more men than women in chair and full professor positions, and significantly more women than men in associate and assistant professor positions in 2022. There was no significant difference between the number of women and men in chief roles in 2022.

**Figure 2.**
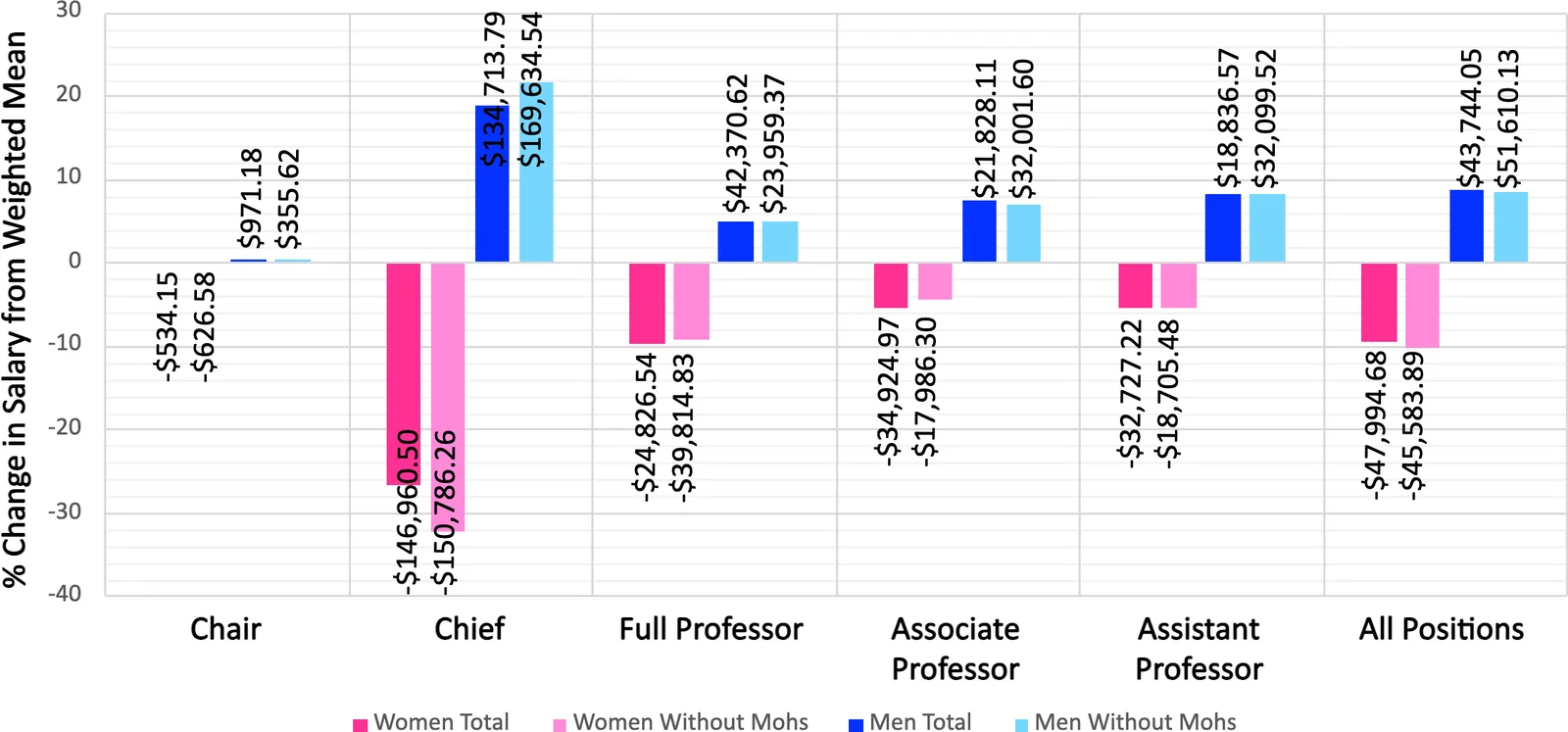
Percent change from weighted mean salary for women and men in dermatology academic leadership roles in 2022. Salary data from the Association of American Medical Colleges (AAMC) Faculty Salary Report 2022 [3]. A weighted mean salary was calculated for each position—compared between men and women—and the percentage change from the mean is displayed. Women reported salaries below the weighted mean, while men reported salaries above it in all dermatology academic leadership roles in 2022. For chair positions, the percent change for women (–0.15%) and men (0.08%) is too small to be visualized. All currency amounts are in US dollars.

These trends show increased female representation within academic ranks in dermatology relative to previous work [5,6] and increased female associate professors with a persistent and increased pay gap relative to prior AAMC surveys (2013‐2018) [6]. To better understand these trends, Goldin [1,7] highlights different types of job/role trajectories: linear with long continuous hours and nonlinear with more flexibility. Goldin [7] describes “nonlinear” occupations as those offering flexibility but with reduced wage progression—a pattern consistent with dermatology’s structure. Despite higher earnings enabling care services, female physicians still disproportionately take time off for family-related absences [1], leading to missed leadership opportunities, potentially explaining why female representation drops higher up dermatology’s leadership ladder. Given the time required for late-career promotions, we anticipate more women chairs and full professors in the coming decade. Despite differing representations among roles, women still earn less than men in all academic dermatology positions, which is consistent with the wage gap Goldin describes. This trend may reflect women’s diverse roles but underscores a broader pattern of pay inequality within dermatology leadership.

Encouraging wage equity should occur at national, institutional, and personal levels. Nationally and specific to this survey, the AAMC could enhance the questions exploring what information was available during the contract negotiation and how family caretaking responsibilities impact career advancement. Individuals can engage with other national associations, such as the American Medical Women’s Association, the Women’s Dermatologic Society, and the American Academy of Dermatology, through their diversity/equity initiatives to explore mentorship opportunities, advocacy roles, and professional advancement. Institutionally, focus groups could evaluate how parental leave policies, flexible work (ie, teledermatology), mentorship programs, and leadership training impact promotion. Stay interviews could help identify department-specific areas of improvement, and assigning merit toward collaboration in publications could help enhance the academic development of women who are faculty members [8]. Focus groups could then be best informed on mitigating systemic bias against women, although Goldin [1] highlights that discrimination is not the primary driving force of the gender wage gap.

This editorial highlights persistent gender pay differences among academic dermatologists. We urge the AAMC to explore these differences through more nuanced survey questions in the faculty salary report. By integrating Goldin’s insights, we aim to promote an equitable environment in dermatology, recognizing the complex interplay between gender, compensation, and career progression.

## Abbreviations

AAMC: Association of American Medical Colleges
